# The calcified lung nodule: What does it mean?

**DOI:** 10.4103/1817-1737.62469

**Published:** 2010

**Authors:** Ali Nawaz Khan, Hamdan H. Al-Jahdali, Carolyn M. Allen, Klaus L. Irion, Sarah Al Ghanem, Shyam Sunder Koteyar

**Affiliations:** *North Manchester General Hospital, Pennine Acute NHS Trust, Manchester, Saudi Arabia*; 1 *King Saud University for Health Science, King Adulaziz Medical City, Riyadh, Saudi Arabia*; 2 *The Cardiothoracic Centre Liverpool NHS Trust, The Royal Liverpool University Hospital, UK*

**Keywords:** Benign pulmonary nodules, malignant pulmonary nodules, calcification

## Abstract

The aim of this review is to present a pictorial essay emphasizing the various patterns of calcification in pulmonary nodules (PN) to aid diagnosis and to discuss the differential diagnosis and the pathogenesis where it is known. The imaging evaluation of PN is based on clinical history, size, distribution and the gross appearance of the nodule as well as feasibility of obtaining a tissue diagnosis. Imaging is instrumental in the management of PN and one should strive not only to identify small malignant tumors with high survival rates but to spare patients with benign PN from undergoing unnecessary surgery. The review emphasizes how to achieve these goals. One of the most reliable imaging features of a benign lesion is a benign pattern of calcification and periodic follow-up with computed tomography showing no growth for 2 years. Calcification in PN is generally considered as a pointer toward a possible benign disease. However, as we show here, calcification in PN as a criterion to determine benign nature is fallacious and can be misleading. The differential considerations of a calcified lesion include calcified granuloma, hamartoma, carcinoid, osteosarcoma, chondrosarcoma and lung metastases or a primary bronchogenic carcinoma among others. We describe and illustrate different patterns of calcification as seen in PN on imaging.

Calcification in a pulmonary nodule (PN) on imaging indicates a high probability that the lesion is benign. But not all calcified PN are benign and the differential considerations include a primary central lung carcinoid, metastasis and a primary bronchogenic carcinoma. The widespread use of computed tomography (CT) has increased the sensitivity of detecting calcification in malignant tumors[1] [Figure 1]. Radiological demonstration of calcification in lung cancers is uncommon but when encountered may lead to misdiagnosis. Amorphous, punctate, and reticular patterns of calcification have been described in lung cancer. Malignant tumors may engulf a pre-existing granuloma, or tumor necrosis can manifest as tumor dystrophic calcification. Calcification in a mucinous adenocarcinoma may occur as a primary phenomenon. In a malignant PN, calcification appears in the form of larger lesions and is usually stippled or eccentric. To classify calcification in a benign PN certain criteria need to be fulfilled. Benign calcification should encompass over 10% of the PN and calcification should be central, diffuse, popcorn type or laminated.[2] To complicate matters malignant nodules may mimic the appearances of benign calcified granulomas; typical examples are metastases from osteogenic sarcoma or chondrosarcoma. Six different patterns of calcification in a PN are known: (I) central dense nidus (II) diffuse solid (III) laminated (IV) popcorn (V) punctate and (VI) dendriform. The first three types follow granulomatous processes. Popcorn-like calcification typically occurs in a pulmonary hamartoma (PH). Diffuse, central, laminated or popcorn calcifications are considered benign and usually seen in granulomas and PH. All other patterns of calcification should not be regarded as a sign of benignity.[1,3–6]

**Figure 1:**
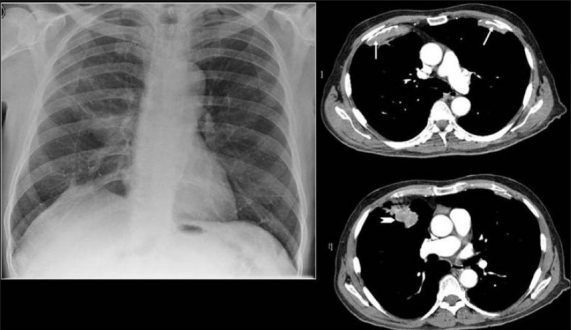
A conventional radiograph of patient presenting with hemoptysis and weight loss shows a right hilar mass but no calcification is apparent. Two sections of axial CT scans show calcified pleural plaques (white arrows) due to previous asbestos exposure complicated by a bronchogenic neoplasm (arrow head)

Calcification in PN is optimally visualized at low-kilovoltage radiography or chest fluoroscopy. High-resolution CT (HRCT) is significantly more sensitive than a conventional chest X-ray for quantitative assessment of intratumoral calcification.[4,7–9]

If calcification is not apparent on HRCT, its presence can sometimes be inferred from CT attenuation values determined with CT densitometry. Attenuation value of 200 HU or higher is generally regarded as evidence of calcification. CT densitometry has been shown to have limited value when assessing spiculated nodules and its sensitivity (66%) and specificity (98%) for benign disease are not optimal.[4,8,9]

Differential diagnosis of diffusely distributed small calcified nodules includes infections, lung metastases, chronic pulmonary hemorrhage, pneumoconiosis, deposition diseases and idiopathic disorders such as pulmonary alveolar microlithiasis.[10] It is imperative that before embarking on the workup of a high-density nodule that an extrinsic thoracic wall lesion is excluded [Figure 2].

**Figure 2:**
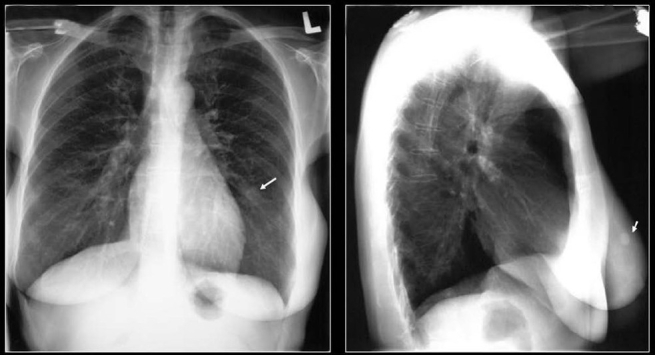
It is always important to establish as to whether a PN is not from an abnormality outside the lungs. Here, a calcified nodule within the breast (arrow) mimics a PN

Approach to diagnosis of calcified pulmonary nodule:

To aid diagnosis PNs may be classified as small or large, single or multiple and within this frame work high parenchymal lung densities should also be considered in the differential diagnosis. The prevalence of calcified lung cancers identified on conventional chest radiographs is said to be 1%. Thus, calcification in PN does not guarantee benignity.[1,3,11–15] However, radiographs of resected of lung cancers specimens have shown calcific deposits in 16% specimens.[14] To distinguish benign from malignant PN a close attention to the pattern of calcification is required. Benign PNs typically show dense central, laminated or diffuse calcifications a pattern that is said to virtually exclude malignancy. However, punctate or eccentric calcification considered indicative of malignant lesions are occasionally seen in benign lesions.[15] Modern, CT scanners have a much higher sensitivity with increased prevalence of 6%-10% calcification in lung cancers.[1,3,13–15] Calcification pattern in the primary lung cancer may have a varied appearance such as isolated flecks, amorphous, punctate and reticular patterns. Some authors have found no correlation between the tumor histology and pattern of calcification while others have reported intratumoral calcification in 23% of small cell carcinomas.[1,3,13] Round or oval lung nodules that have diffuse or focal calcification evident on HRCT that measures 10-30 mm are classified as large.[11,16] Calcified nodules may present as single PN, multiple large PN or diffuse small calcified pulmonary nodules. On imaging distinction has to be made from other noncalcific lung densities.

## Single Large Pulmonary Nodules

### Pulmonary hamartoma

**Figure 3:**
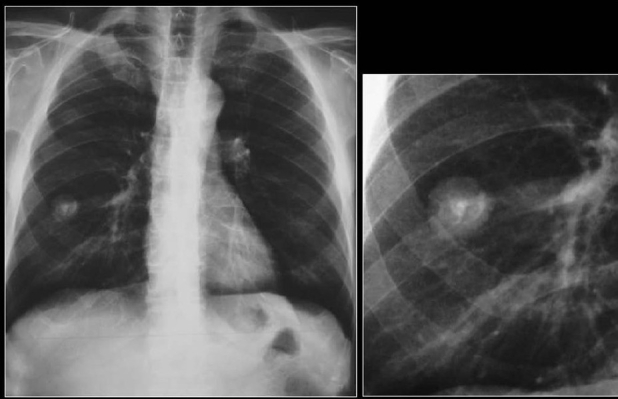
A chest radiograph shows a fairly well-defined PN in the right mid zone associated with a central nidus and a laminated calcification in a pulmonary hamartoma

**Figure 4:**
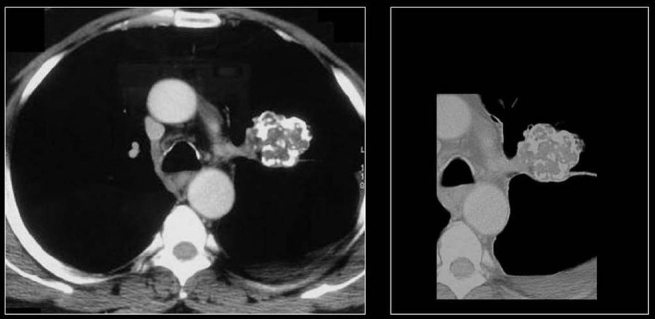
An axial CT scan just above the hila shows a large central PN with popcorn calcification in a hamartoma

PH is the most common benign lung tumor, composed of tissues that are normally present in the lung, including fat, epithelial tissue, fibrous tissue and cartilage. However, they exhibit disorganized growth. Most PH do not cause symptoms and have no malignant potential. PH may mimic a bronchogenic carcinoma; therefore, accurate imaging interpretation is important.[17,18] On chest radiographs, PHs characteristically appear as sharply outlined round/oval lobulated solitary pulmonary nodules, 4 cm or less; they may show varying patterns of calcification as discussed above [Figure 3]. Popcorn calcification is virtually diagnostic [Figure 4]. Calcification that is detectable on plain radiographs is reported to occur in 10%-15% of patients. Any lobe of the lung may be involved, and cavitation is extremely rare. In rare instances, central tumors may cause bronchial obstruction with distal obstructive pneumonitis, bronchiectasis and progressive peripheral lung destruction. Serial chest radiographs may demonstrate no or slow growth. A rare rapid growth has been described, thus posing difficulty in differentiation from a bronchogenic carcinoma.[19]

**Figure 5:**
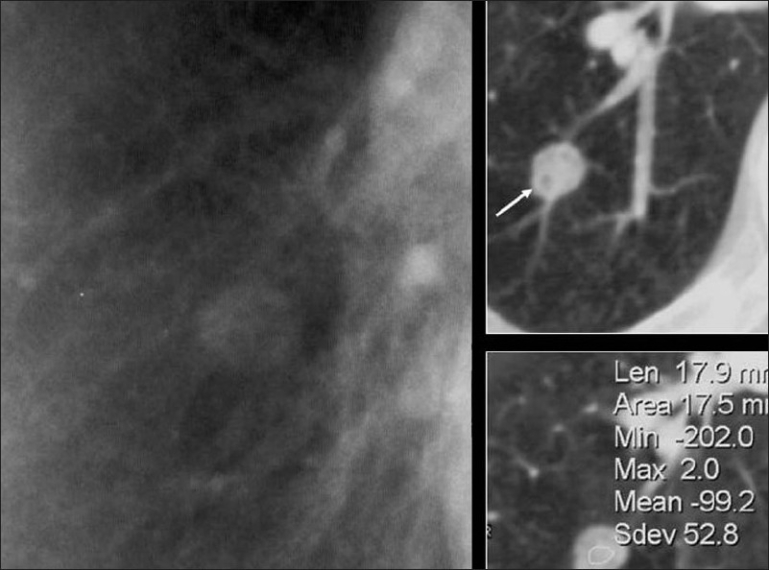
A blown-up image from a chest radiograph and axial CT scans showing low attenuation areas (arrow) within the PN due to fat virtually diagnostic of a hamartoma

The appearances of PH on CT are similar to those on chest radiographs. CT criteria for PH included a diameter of 2.5 cm or less, a smooth edge, and focal collections of fat or fat alternating with areas of calcification. PH with no detectable calcium is not usually diagnosed by means of CT.[4] However, thin sections on CT allow for more detailed evaluation of the internal architecture and morphology of lesions. On HRCT, fat is identified in 34%-50% of PH, and calcification, in 15%-30% [Figure 5]. The finding of fat and calcification together is a specific combination for PH, particularly in tumors of diameter 2.5 cm or less. The frequency of calcification increases with increasing tumor size; calcification is found in only 10% of lesions smaller than 2 cm, but the frequency increases to 75% for lesions larger than 5 cm.[20–22] Both calcium and fat are better visualized with CT than with conventional radiographs.[23,24]

When fat is encountered within a PN on CT or magnetic resonance imaging other fat-containing lesions should also be considered. Fat-containing lung lesions include parenchymal and endobronchial lesions such as PH, lipoid pneumonia and lipoma. Endobronchial PH usually appears at CT as a lesion with a smooth edge, focal collections of fat or fat collections that alternate with foci of calcification. Differentiation is made from mediastinal fat-containing lesions such as germ cell tumors, thymolipomas, lipomas and liposarcomas. Fat may also herniate into the chest at several locations. CT reformatting can add confidence to the diagnosis when fat herniation is suspected.[25]

An air cleft on the side or the inside is characteristic of PH. Pulmonary artery branches connect beyond half of PH. This finding suggests close relations in the bronchus along the artery. It is important that there is no connection of the pulmonary vein, to differentiate it from a bronchogenic carcinoma.[26,27]

Magnetic resonance imaging may further characterize a discrete pulmonary nodule that demonstrates neither fat nor calcification on CT, in detecting a typical cleft-like structure in a PH providing further diagnostic confidence.[26,27]

A majority of PH represent as solitary PN. Peripheral tumors are usually simply observed after the definitive diagnosis; central tumors may be excised. The prognosis is excellent. With atypical PH fast frozen section at surgery is critical to acquire accurate pathological diagnosis. Definite diagnosis and the treatment can be achieved by surgical resection with minimal morbidity. Due to potential trend of recurrence, patients with PH should be submitted to a complete evaluation and a regular follow-up.[28–32] About 20% of (mainly large size) PH have uptake characteristics suggesting malignancy on positron emission tomography/CT.[33]

### Calcified granuloma

**Figure 6:**
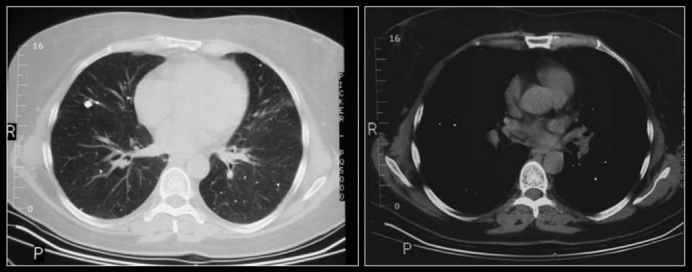
Axial CT scans shows multiple small calcific PNs due to old healed histoplasmosis

A variety of infections can elicit a granulomatous tissue response and result in a multitude of intrathoracic dystrophic calcifications. A prime example is *Histoplasma capsulatum*, which give rise to a variety of calcified intrathoracic calcific deposits including calcified mediastinal lymph nodes, broncholithiasis, mediastinal granuloma and solitary or multiple intrapulmonary calcified histoplasmomas[34] [Figure 6].

Besides larger histoplasmomas, there may be multiple, small, diffuse, calcified pulmonary nodules in asymptomatic patients. This type of calcification most probably results from either hematogenous or bronchogenic dissemination of *H. capsulatum.*[35]

Hypercalcemia secondary to granulomatous infections or supplemental vitamin D is a rare occurrence but may contribute to calcification of intrathoracic granulomas.[36–38]

Calcified lung granulomas secondary to *Coccidioides immitis* infection is a rare occurrence. Dystrophic calcification may occur in healing coccidioidal granulomas.[39]

**Figure 7:**
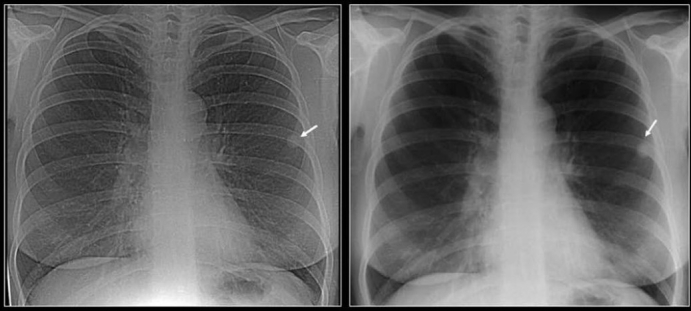
Two chest radiographs 5-years apart showing a high-density solitary pulmonary nodule remaining unchanged over a 5-year period. One of the most reliable imaging features of a benign lesion is as a benign pattern of calcification and periodic follow-up with CT showing no growth for 2 years. The high density of the well-defined nodule suggest that this is calcified granuloma and no further follow-up is indicated except in patients with calcium producing tumors such as a primary osteosarcoma

**Figure 8:**
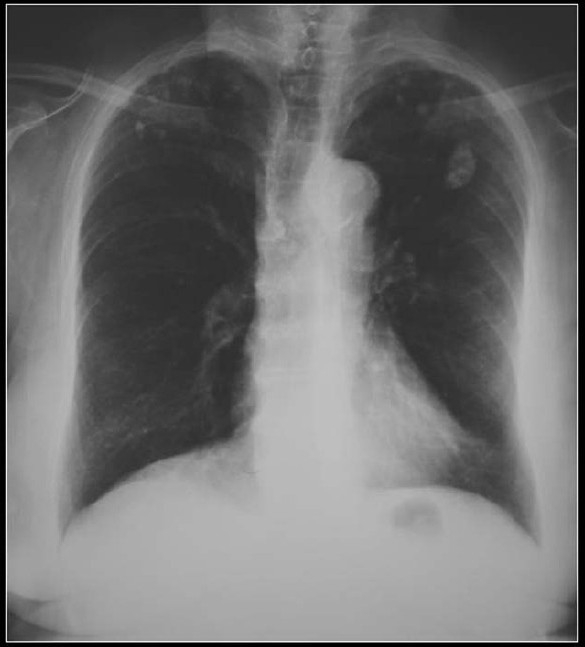
A chest radiograph shows calcification at both the lung apices associated with a right lower paratracheal calcified lymph node due to healed tuberculosis

**Figure 9:**
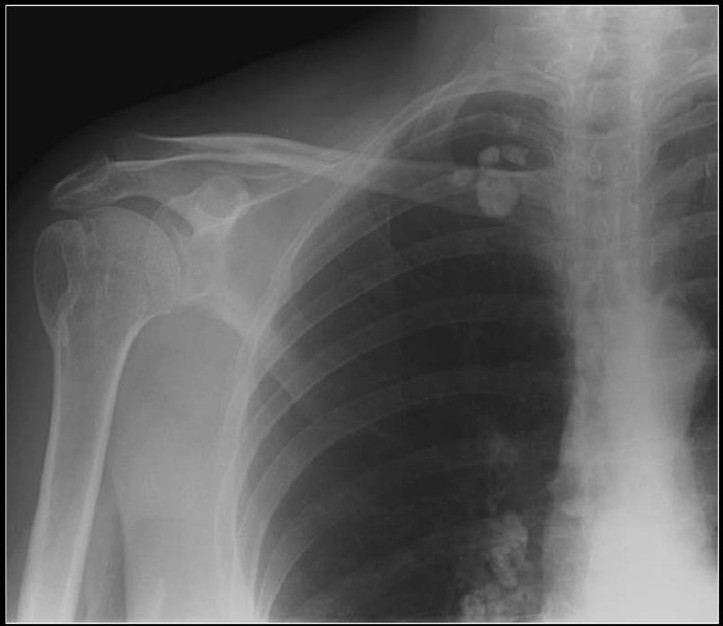
A radiograph taken for the right shoulder shows old healed granulomatous disease at the right apex of the lung associated with calcified right hilar lymph nodes

**Figure 10:**
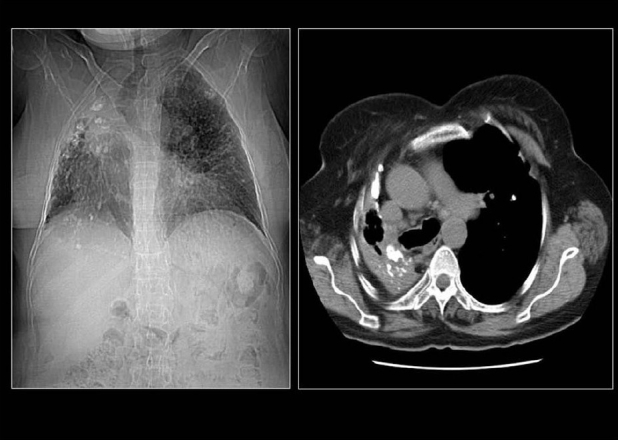
A chest radiograph and CT showing features of old healed TB. Note the loss of lung volume/fibrosis in the right upper zone and the associated pleural calcification due to a previous tuberculous empyema. Calcific granulomas are also noted in the left apical region

Tuberculosis (TB) is a common cause of intrathoracic calcifications. Most calcific deposits in TB are dystrophic and may present as parenchymal granulomas, mediastinal lymph nodes, and fibronodular areas of lung involvement [Figures 7–10]. Diffuse nodular calcification of the lungs may be the result of a hematogenous infection. Re-evaluation of value of HRCT for controversial lesions is needed. It has been shown that patients with TB can develop hypercalcemia caused by excessive production of endogenous 1,25 vitamin D.[40] Rarely, sarcoidosis may produce multiple micronodular calcifications, radiographically similar to pulmonary alveolar microlithiasis (PAM).[41–42] However in sarcoidosis, calcification is within epitheloid granulomas, while in PAM, there are distinct intra-alveolar microliths.[43]

### Primary lung cancer

**Figure 11:**
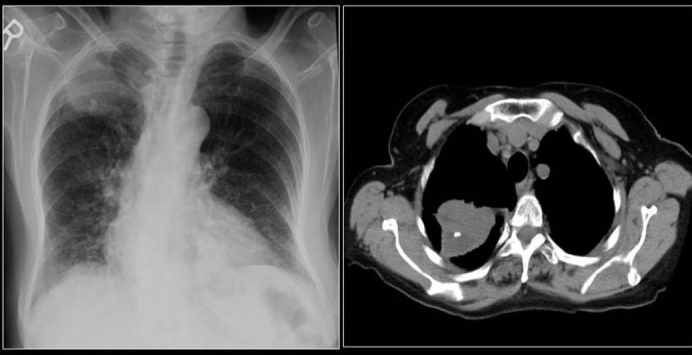
A chest radiograph and axial CT scan shows a dense nidus of central calcification in an adenocarcinoma of the lung

**Figure 12:**
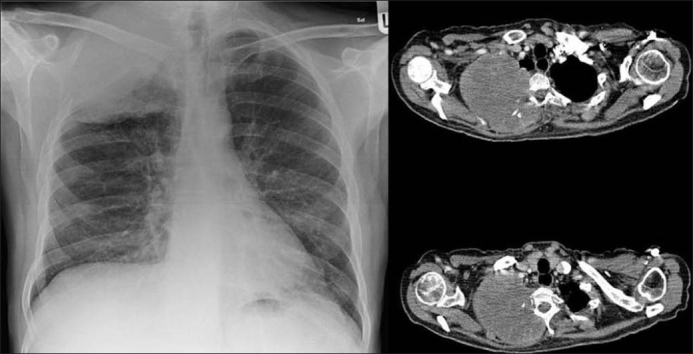
Bronchial neoplasms in contact with the thoracic wall may invade ribs and adjacent vertebrae and engulf destroyed pieces of bone and thus mimic intratumoral calcification as in this pancoast tumor

**Figure 13:**
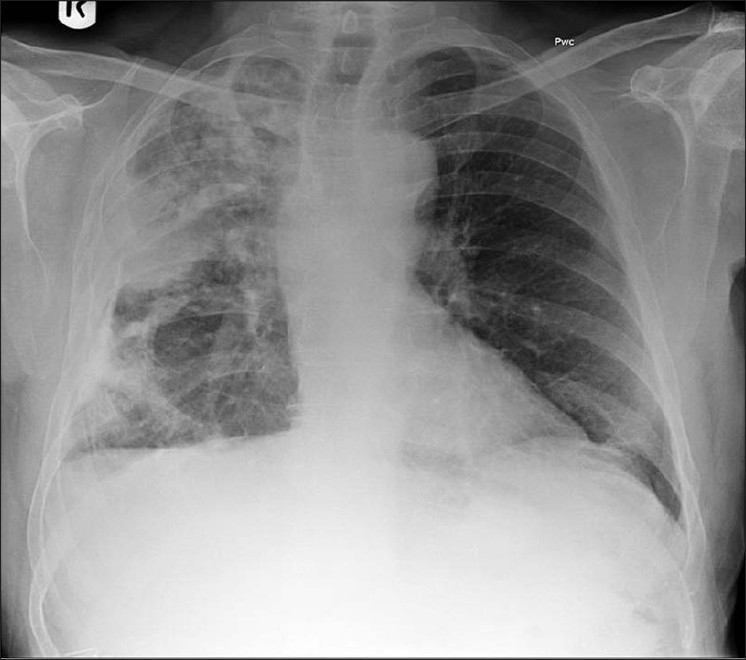
A chest radiograph of a patient with history of asbestos exposure shows multiple right-sided pleural plaques some calcified and a large pleural based PN raising the suspicion of a mesothelioma (See Figure 14)

**Figure 14:**
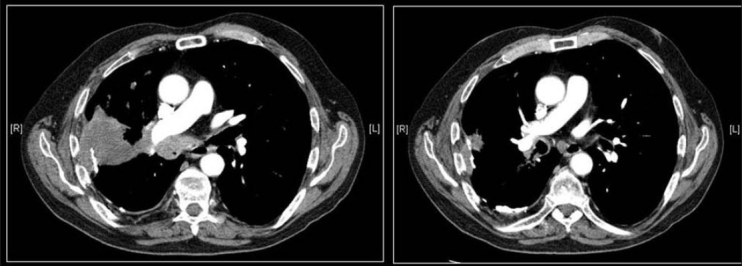
Axial CT scans of the same patient as in Figure 13 shows a pleural-based mass with underlying pleural calcification. A CT-guided biopsy revealed a small cell lung cancer

High CT numbers of solitary nodules do not ensure a benign lesion.[44] A review of CT scans of 353 patients found visualization of calcification within lung cancer rare but when discovered they caused confusion and misdiagnosis. In 6% of the patients, CT scans revealed calcification. The tumor size was variable included tumors larger than 5 cm or greater and 2 cm or smaller. Cell types of the tumors included small cell carcinoma, squamous cell carcinoma, adenocarcinoma and undifferentiated carcinoma. Patterns of calcification were punctate, amorphous and reticular in decreasing order of frequency. Extent of tumor calcification and distribution such as central, peripheral or diffuse did not correlate with cell type or size of the lesion[3] [Figure 11]. Visualization of calcium on chest radiographs and CT scans do not alone excludes the diagnosis of bronchogenic carcinoma.[3,14] Another study of 500 CT examinations revealed calcification in 10% of lung cancer, calcification did not predict histological subtype, but found calcification more common large, central tumors.[1] Bronchial neoplasms in contact with the thoracic wall may invade ribs and adjacent vertebrae and engulf destroyed pieces of bone and thus mimic intratumoral calcification as in this Pancoast tumor [Figure 12]. Peripheral bronchogenic tumors complicating asbestos lung disease are often associated with underlying calcified pleural plaques [Figures 13–14].

The prevalence of calcification in large cell neuroendocrine carcinoma (LCNEC) is similar to that of other lung cancers. The mechanism of the intratumoral calcification in LCNEC cases is speculated to be dystrophic calcification.[45]

Calcification in primary lung cancers has been proposed to have different mechanisms ranging from engulfment of a calcific scar tissue, degenerated bronchial cartilage and granulomatous process engulfing the tumor to dystrophic calcification in areas of tumor necrosis. Calcification may also develop as sequelae to chemotherapy or in association with hypercalcemia. Calcium deposition may also occur as a result of a secretary function of the carcinoma.[3,14,46,47]

### Carcinoid

**Figure 15:**
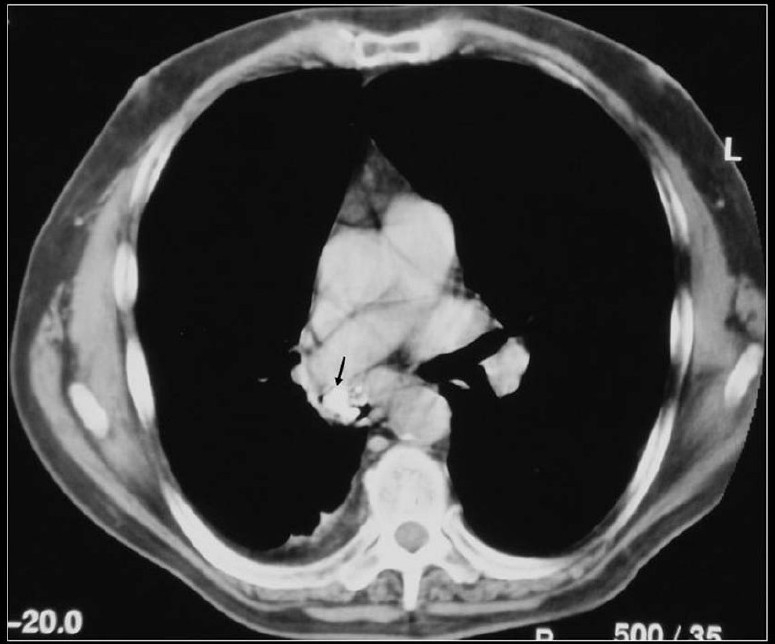
Axial CT scan shows a central pulmonary carcinoid associated with dense amorphous calcification (arrow)

**Figure 16:**
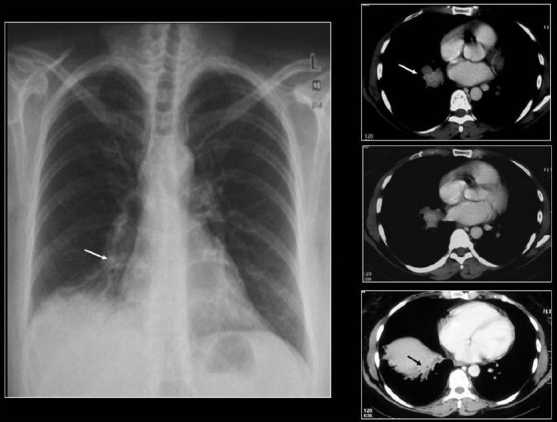
Bronchial carcinoid shown as dense nodule at the lower pole of the right hilum on the chest radiograph (arrow). On axial CT scans there is nonuniform density of the PN (white arrow), which revealed calcification on the resected specimen. Note the subsegmental atelectasis distal to the tumor (black arrow)

Lung carcinoid tumors are often misdiagnosed as a carcinoma. The possibility of carcinoid should be considered in a young nonsmoker with lung tumor where bone or calcification is present in the tumor [Figures 15 and 16]. Carcinoid tumors is predominantly central calcification has been reported in 8%-35% of the cases.[48,49]

### Intrathoracic sarcomas

**Figure 17:**
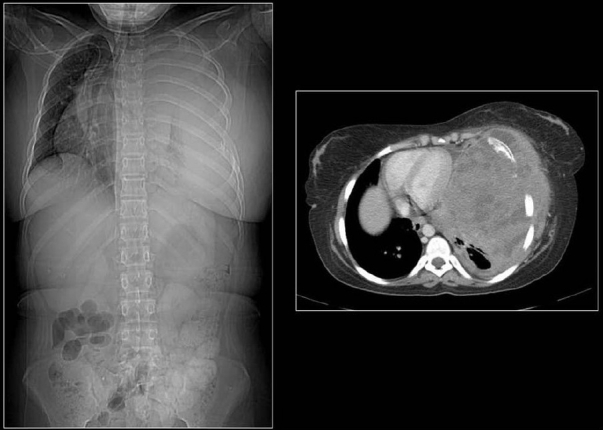
A Ewing's sarcoma of a rib invading the left pleura and left lung showing small high-attenuation nodules due to entrapped fragmented bone

Primary intrathoracic sarcomas may originate in the lung, mediastinum, pleura and chest wall. Classification is based on histological features. Calcification can occur in angiosarcoma, leiomyosarcoma and rhabdomyosarcoma. Several variants of mesotheliomas are difficult or impossible to differentiate from sarcomas. Sarcomatous mesothelioma is a rare variant that may have osteogenic properties. Sarcomas that primarily arise in the thoracic wall include Ewing sarcoma, primitive neuroectodermal tumor, chondrosarcoma, malignant fibrous histiocytoma, osteosarcoma, synovial sarcoma and fibrosarcoma [Figure 17]. Primary intrathoracic sarcomas are usually large tumors at presentation and are generally seen as heterogeneous masses; however, a wide spectrum of radiological findings have been described, including solitary pulmonary nodules, central endobronchial tumors and intraluminal masses within the pulmonary arteries. Intrathoracic sarcomas are usually indistinguishable on imaging. However, careful attention to the location of the tumor, its clinical presentation and features such as calcification within the mass and rib destruction, may provide clues as to the origin and type of sarcoma. For example, a mass with a calcified matrix is more likely a chondrosarcoma or osteosarcoma, and a pulmonary artery mass is likely to be a leiomyosarcoma.[50,51]

### Metastases

**Figure 18:**
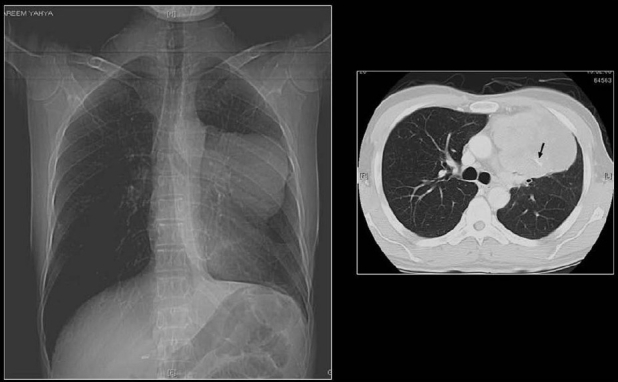
A mediastinal/lung metastasis from a soft tissue sarcoma of the thigh showing a linear calcific density (arrow) confirmed as calcification on resected specimen

**Figure 19:**
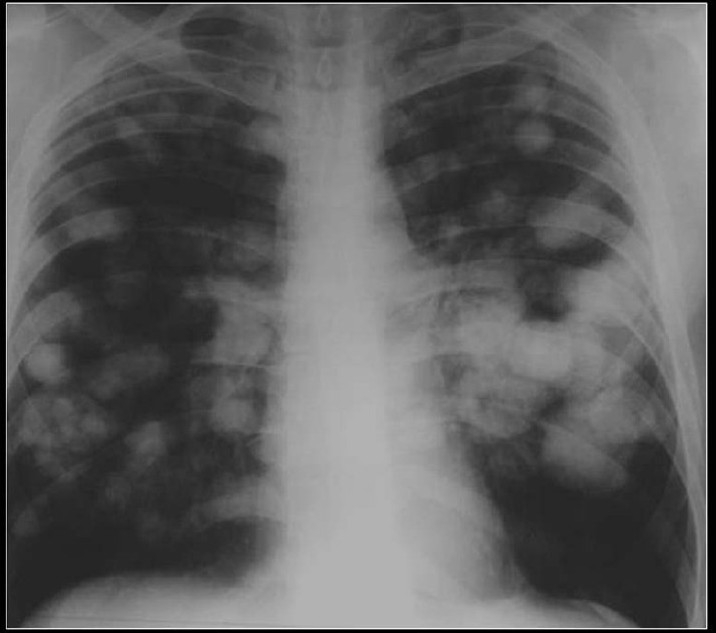
A chest radiograph showing calcified metastases from an osteogenic sarcoma. Note that the density of the tumors and the skeletal tissues is similar

**Figure 20:**
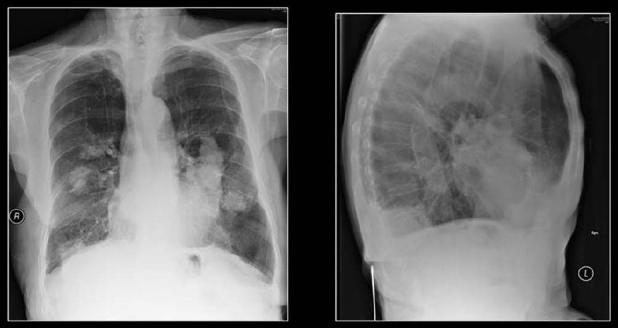
PA and lateral chest radiographs showing multiple high-density lung masses in a patient with a nonmucinous adenocarcinoma of the sigmoid colon (see CT scans in Figure 21)

**Figure 21:**
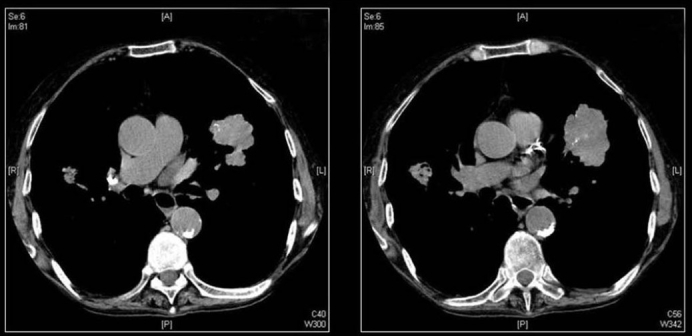
CT scans of the same patient as in Figure 20 shows multiple calcified metastases from a non-mucinous adenocarcinoma from sigmoid colon confirmed on CT guided needle biopsy

**Figure 22:**
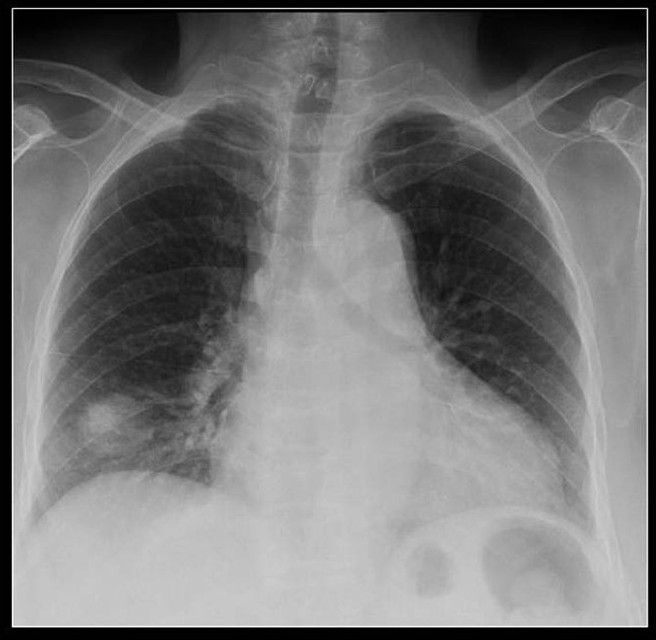
A chest radiograph showing a large PN at the right lung base with central high density due to calcification in a metastatic deposit from a leiomyosarcoma of the uterus

**Figure 23:**
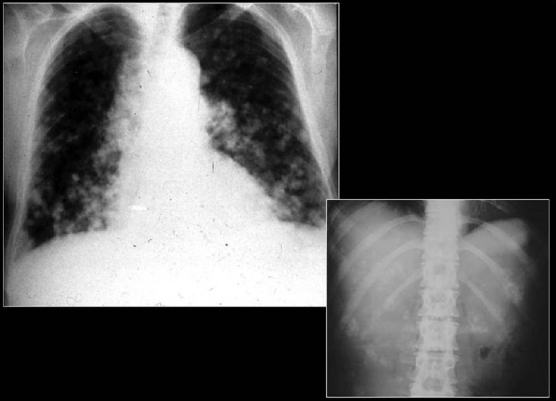
A PA chest radiograph of a patient with calcified metastases from a medullary carcinoma of the thyroid. A radiograph of the upper abdomen on the same patient showing calcified metastases to the liver

Lung metastases may be single but more often multiple. Most calcified pulmonary metastases are from primary sarcomas such as osteogenic, chondrosarcoma, synovial sarcomas, giant cell tumor, malignant mesenchymoma and fibrosarcoma of the breast. Metastases from other primary carcinomas that may calcify are papillary and mucinous adenocarcinomas [Figures 18–22]. Occasionally, pulmonary metastases from a medullary carcinoma of the thyroid may calcify [Figure 23].

Calcified pulmonary metastases have been reported on CT images from a breast malignant cystosarcoma phylloides. Histological examination of the pulmonary masses revealed malignant spindle cells with osteoid and cartilage components in the cellular stroma.[52]

The metastases of a number of other tumors have calcified after anticancer therapy. A variety of mechanisms are involved in the genesis of calcified lung metastases: bone formation in tumor osteoid, calcification and ossification of tumor cartilage, dystrophic calcification and ossification of tumor cartilage, dystrophic calcification and mucoid calcification.[53]

### Calcifying fibrous pseudotumor

Pincard *et al* have described 3 cases of “calcifying fibrous pseudotumor” (CFPT) in whom CT scans revealed pleural-based nodular masses with central areas of calcifications. These lesions were limited to the pleura with no lung parenchymal involvement. Histology of these masses revealed circumscribed, but unencapsulated masses of hyalinized collagenous fibrotic tissue interspersed with lymphoplasmacytic infiltrates and calcifications. CFPT is distinct from a fibrous tumor of pleura, calcified granulomas, calcified pleural plaques and chronic fibrous pleuritis.[54]

### Intralobar pulmonary sequestration

**Figure 24:**
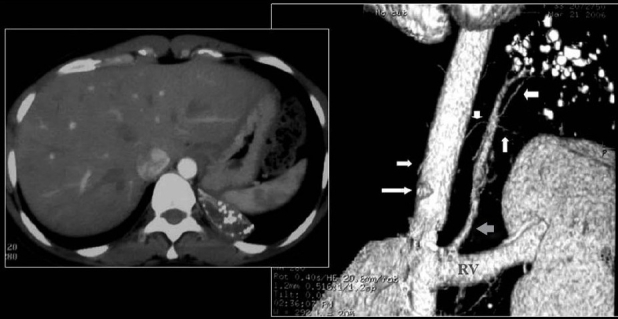
A rare calcified intralobar sequestration; the arterial supply and venous drainage is elegantly shown by the CT angiography (right) note that the arterial blood supply is arising from the celiac axis (white arrows) and venous drainage is via the left renal vein (gray arrow)

The radiological diagnosis of intralobar pulmonary sequestration (ILPS) is based on the identification of a feeding systemic artery on CT. Radiographically demonstrated calcification is rare and has been described only three times.[55] We illustrate a rare case of our own [Figure 24].

## Multiple Large Calcified Nodules or Masses

The main diagnostic considerations are PH, calcified pulmonary metastases, primary lung tumors (rare), pulmonary chondromas, carcinoid, amyloidosis, calcified hyalinizing granulomas, necrobiotic nodules and progressive massive fibrosis.[10–11]

### Amyloidosis

Respiratory involvement can be demonstrated in 50% of patients with primary amyloidosis on pathological grounds although radiological depiction is rare. Pulmonary amyloid may present as tracheobronchial, nodular or diffuse pulmonary infiltration. The HRCT findings in the diffuse parenchymal form include small well-defined 2-4 mm nodules, abnormal reticular opacities, interlobular septal thickening, and subpleural confluent consolidations. The focal nodular form may be solitary or multiple, which appear rounded or lobular masses with well-defined border. Approximately 50% of nodules calcify or ossify.[56]

### Hyalinizing granulomas

Pulmonary hyalinizing granulomas (PHG) are probably related to a chronic immune reaction to endogenous or exogenous antigens or infectious agents such as *H. capsulatum or Mycobacterium* organisms, and said to occur in individuals predisposed to marked scar formation.[10–11] On imaging PHG appear as well-marginated solitary or multiple nodules varying from a few millimeters to 15 cm in size.[10–11]

### Progressive massive fibrosis

**Figure 25:**
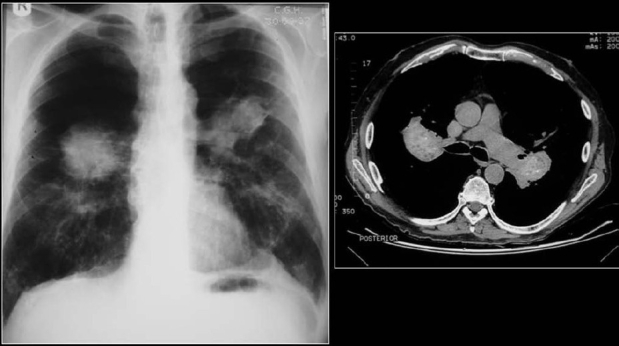
Chest radiograph and an axial CT scan shows calcification PMF in a coal miners lung

Progressive massive fibrosis (PMF) is associated with either silicosis or coal miners' pneumoconiosis or presents as an area of mass-like consolidation on chest radiograph and CT often associated with lung parenchymal scarring and adjacent bullae, usually in the upper lobes. The mass may present as a conglomerate of masses usually oval with irregular borders. PMF is usually bilateral, but a unilateral mass may occur and be confused with cancer. PMF is always associated with small background nodules well depicted on HRCT. Punctate calcification in PMF is often seen on CT[10–11] [Figure 25].

### Metastatic pulmonary calcifications

**Figure 26:**
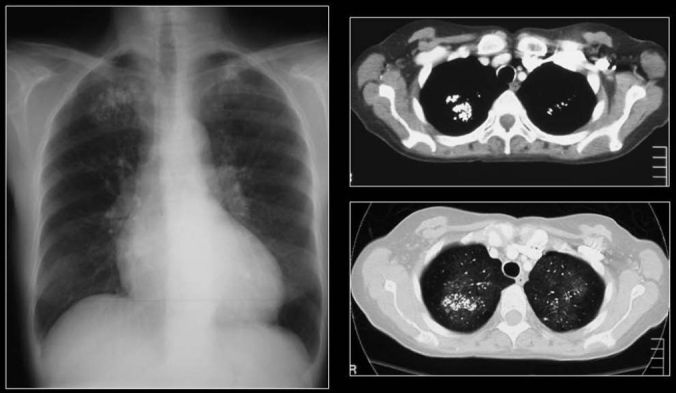
Metastatic calcification in the lungs in a patient with chronic renal failure

Metastatic pulmonary calcification usually occurs in normal pulmonary parenchyma (alveolar walls, bronchi and blood vessel walls), the kidney and the stomach. Such calcification is secondary to abnormal calcium metabolism without any prior soft tissue damage. The predisposing factors for this condition include chronic renal failure, hypercalcemia such as in primary and secondary hyperparathyroidism, chronic renal failure, sarcoidosis, IV calcium therapy, multiple myeloma and massive osteolysis caused by metastases and increased tissue alkalinity. The most common imaging features are poorly marginated nodular opacities in the upper zones of the lungs. A frequent associated finding is calcification in the vessels of the chest wall.[10–11,57–60] Metastatic pulmonary calcification has also been reported in a patient with hypercalcemia due to hyperparathyroidism who recovered from pneumonia with sepsis and whose HRCT images demonstrated localized parenchymal airspace calcification that was limited to the lower lobes[61] [Figure 26].

### Miscellaneous non-neoplastic lung tumors

Many rare non-neoplastic lesions of the lung may mimic lung cancer; these include inflammatory pseudotumor (inflammatory myofibroblastic tumor), placental transmogrification of lung, alveolar microlithiasis and metastatic calcification. Although non-neoplastic, they are nonetheless important to recognize, as their outcome may not necessarily be an innocuous one.[62]

### Carney triad

The Carney triad is syndrome associated with gastric stromal sarcomas, pulmonary cartilaginous tumors and extra-adrenal paragangliomas. The pulmonary neoplasms in the Carney triad are designated as chondromas and are often misinterpreted clinically and pathologically as metastases from the gastric tumors. The pulmonary neoplasms in the Carney triad are well-differentiated benign cartilaginous tumors that are best designated as chondromas and can be differentiated pathologically from pulmonary cartilaginous PH on the basis of the presence of a thin fibrous pseudocapsule, frequent bone metaplasia, and calcification, and also the absence of entrapped epithelium and fat.[63]

## Diffuse Small Calcified Nodules

### Infections

**Figure 27:**
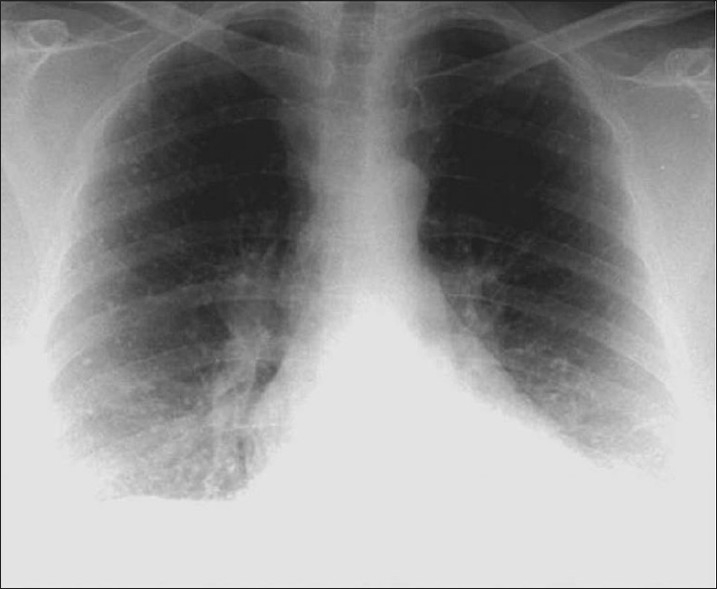
Miliary diffuse calcific nodules in an adult male with a previous history of Varicella pneumonia

**Figure 28:**
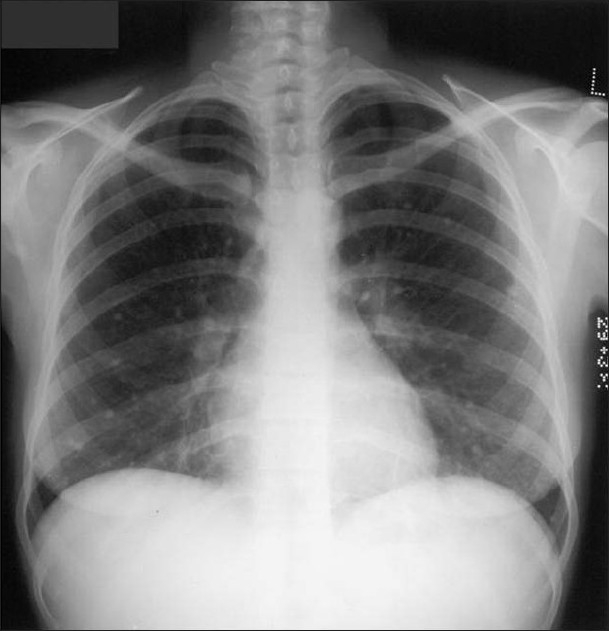
A chest radiograph shows multiple small calcific nodules in an adult female with a past history of Varicella pneumonia

**Figure 29:**
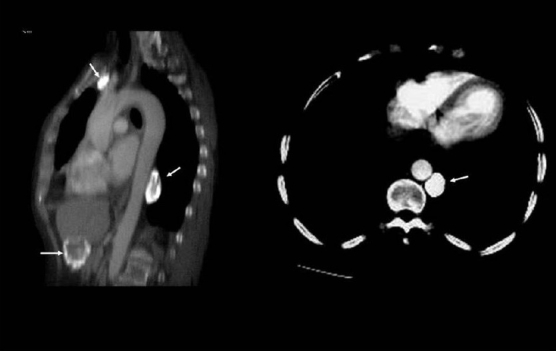
Hydatid cysts do not normally calcify within the lungs; two calcified hydatid cysts in the superior and posterior mediastinum are seen. Note the calcified hydatid cyst within the left lobe of the liver on the coronal CT reconstruction

Small calcified lung nodules are often the result of dystrophic calcification in areas of injured lung.[10] Dystrophic calcification follows caseation, necrosis or fibrosis. Calcified nodules following infections are well defined and often measure 2-5 mm in diameter. Such nodules often follow healed disseminated histoplasmosis and rarely may follow healed miliary tuberculosis. Patients with multiple pulmonary nodular calcifications secondary to tuberculosis or histoplasmosis generally have associated calcified hilar or mediastinal lymph nodes.[10–11] The CT findings of inactive pulmonary tuberculosis include calcified nodules or consolidation, irregular linear opacity, parenchymal bands and pericicatricial emphysema. Underlying histopathology findings of typical and atypical CT findings of tuberculosis are caseating granulomas or pneumonia in the active phase and fibrosis and dystrophic calcification in the inactive phase.[64] Chicken pox pneumonia may cause tiny widespread micronodular calcification with nodules 1-3 mm in diameter as a late sequela [Figures 27 and 28]. There is no associated calcification of mediastinal lymph nodes.[10] Hydatid cysts of the lungs generally do not calcify [Figure 29].

### Hemosiderosis

Idiopathic pulmonary hemosiderosis causes recurrent episodes of alveolar hemorrhage over several years usually in infants and young adults. The end result is pulmonary hemosiderosis that causes centrilobular nodular opacities depicted on HRCT. Calcium is often added to the hemosiderin causing increased density of these nodules on imaging. Similar findings of multifocal calcific nodules can occur in patients with secondary hemosiderosis associated with mitral stenosis.[65]

### Pneumoconiosis

**Figure 30:**
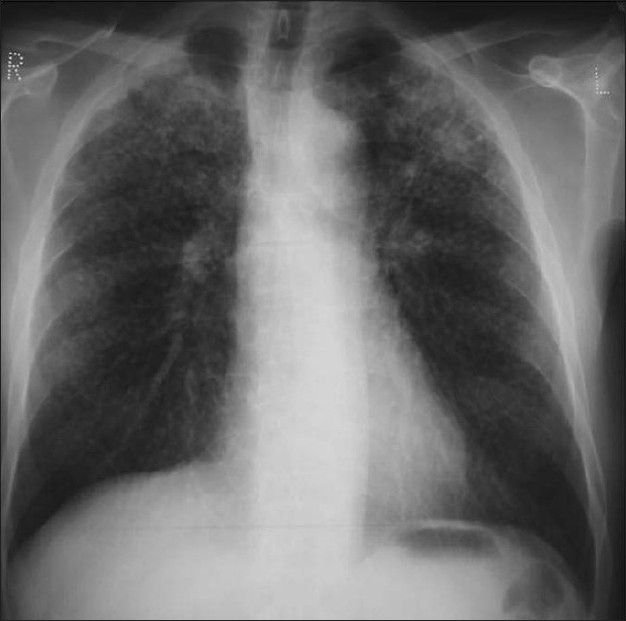
A chest radiograph shows reticulonodular shadowing with bilateral apical lung fibrosis and high density nodules in coal workers pneumoconiosis

**Figure 31:**
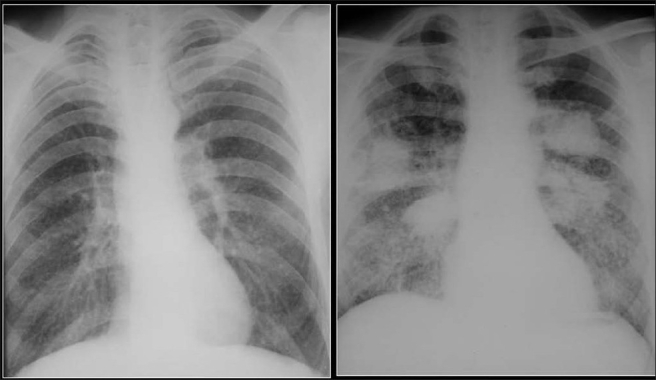
Two chest radiographs from the same Iron ore worker 10 years apart shows fine high-density nodules (left) progressing to PMF 10 years later

**Figure 32:**
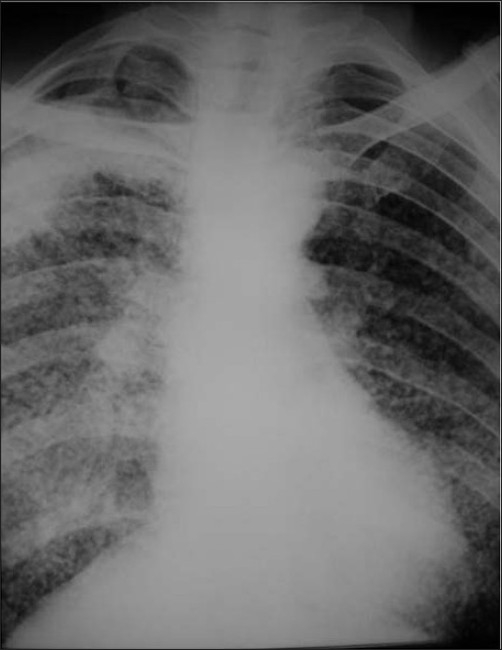
A chest radiograph shows multiple high density nodules due to a lifetime exposure to hematite and silica

**Figure 33:**
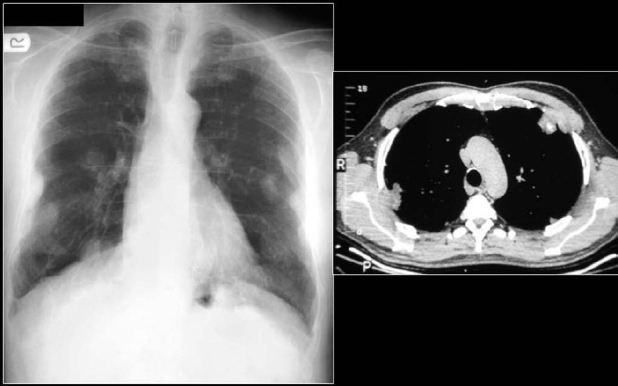
Multiple calcified pleural plaques mimicking PNs on a chest radiographs elegantly depicted on axial CT scan as calcified pleural plaques from previous asbestos exposure

Patients with silicosis and coal miner's pneumoconiosis often develop small (<5 mm) diffuse lung parenchymal calcified nodules, often associated with egg-shell calcification of hilar or mediastinal lymph nodes. HRCT in patients with silicosis and coal workers' pneumoconiosis show diffuse and randomly distributed small well-defined nodules that are most prominent in the middle and upper lung zones.[10,65] Occupational exposure to iron oxide (siderosis), tin oxide (stannosis) and barium dust (baritosis) can cause similar calcific nodules. Stannosis is a condition in which tin oxide is deposited in lung tissue after inhalation. Tin oxide is radiologically visible, although there is no tissue reaction to its presence[10,65] [Figures 30–32]. Pleural plaques may mimic PNs on chest radiographs this confusion does not occur with CT [Figure 33].

### Alveolar microlithiasis

**Figure 34:**
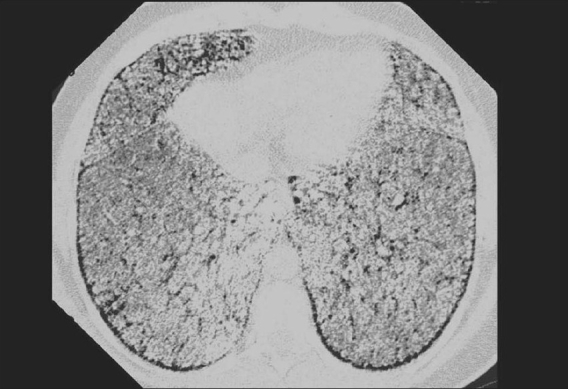
HRCT showing features of pulmonary alveolar microlithiasis. Note the innumerable calcific nodules bilaterally throughout both lungs giving rise to a sand-like appearance

Pulmonary alveolar microlithiasis is a rare idiopathic lung disorder characterized by the intra-alveolar accumulation of microliths of calcium phosphate.[65] Most are an incidental finding on conventional chest radiographs and seen as innumerable tiny calcific densities. HRCT defines the nodules better as tiny sand-like calcified micronodules distributed bilaterally throughout both lungs.[11] Apparent calcification of interlobular septa due to accumulation of calcospherites in alveoli adjacent to the septa is another finding on HRCT, which may be associated with small subpleural cysts[11] [Figure 34].

### Talcosis

Magnesium silicate (Talc) is often used as a filler in a variety of oral medications. Talcosis is the deposition of Talc within the pulmonary arterioles and capillaries. Eventually, multiple small granulomas composed of multinucleated cells containing birefringent crystals evolve. Talcosis is seen drug addicts who use crushed dissolved tablets for intravenous delivery.[66] Initially, HRCT depict numerous high-attenuation nodules measuring less than 1 mm in diameter or a diffuse ground-glass opacities. With time these nodules can become larger confluent masses.[66]

### Acrylic cement embolism

**Figure 35:**
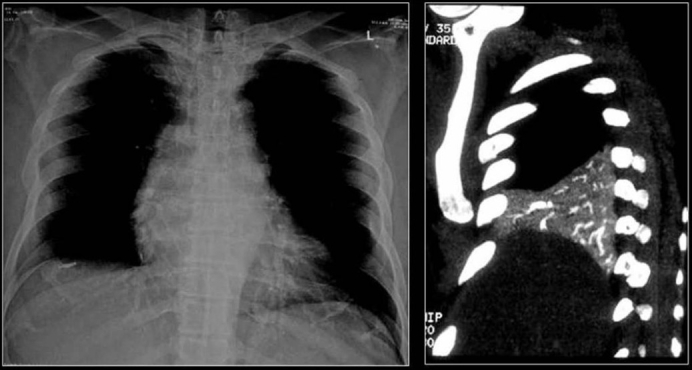
A chest radiograph and coronal reconstruction of CT shows tubular opacities of metallic density due to acrylic cement pulmonary emboli as a complication of vertebroplasty

**Figure 36:**
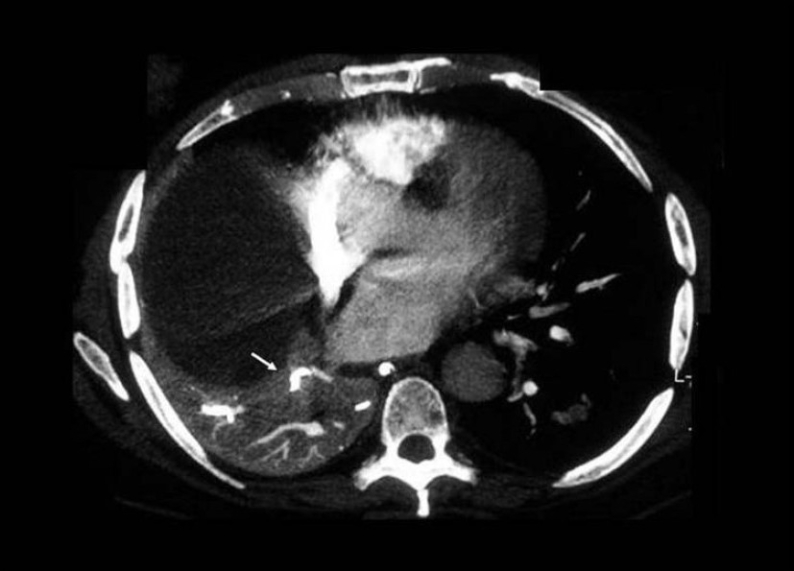
An axial CT of the same patient as in Figure 34 showing tubular opacities of metallic density due to acrylic cement pulmonary embolism as a complication of vertebroplasty

Acrylic cement and sterile barium or tungsten powder opacifier are the elements of a cocktail used to consolidate a collapsed vertebra in a procedure called vertebroplasty. Pulmonary embolism caused by acrylic cement is a rare complication associated with vertebroplasty. The cement reaches the pulmonary artery via the paravertebral venous plexus. Conventional radiographs and CT show multiple radio-opaque tubular areas of increased density corresponding to emboli in the segmental and subsegmental levels of the pulmonary arteries. CT also may depict perivertebral leaks[67] [Figures 35 and 36].

### Dendriform pulmonary ossification

Dendriform pulmonary ossification (DPO) is an uncommon form of diffuse pulmonary ossification that typically affects the pulmonary interstitium in a setting of interstitial fibrosis. DPO is well demonstrated in postmortem examination, and confirmed by microscopy, but rarely diagnosed and virtually never considered clinically. Clinical diagnoses include bronchiectasis and interstitial pneumonitis based on radiographic evidence. Although such calcification often is invisible on chest radiographs, HRCT performed using the appropriate window settings shows tiny calcific opacities in the periphery of the lung.[10,11]

## Diffuse/Focal High-Attenuation Pulmonary Abnormalities

Diffuse high-attenuation pulmonary abnormalities can result from the deposition of calcium or, less commonly, other high-attenuation material such as talc, amiodarone, iron, mercury and barium sulfate.[10] Deposition of calcium salts in tissues or ossification can be secondary to calcification in a collagen matrix (bone tissue) with or without marrow elements.[11]

**Figure 37:**
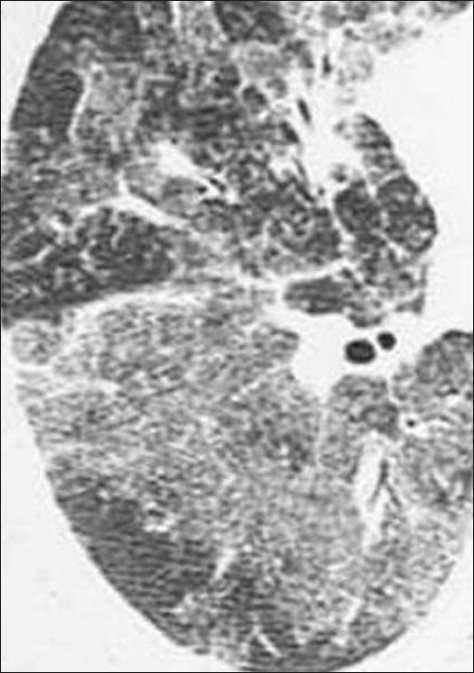
Section of an HRCT showing amiodarone lung. N ote the high density lung, septal thickening and features of interstitial fibrosis

HRCT is highly sensitive in the detection of areas of abnormally high attenuation in the lung parenchyma, blood vessels and airways. However, limited information is available on the HRCT findings of diffuse high-attenuation pulmonary abnormalities and the role of CT in the differential diagnosis[11] [Figure 37].

Disseminated pulmonary ossification can be idiopathic or associated with a variety of pulmonary, cardiac and systemic pathologies. The interstitial dystrophic pulmonary ossification can be focal or diffuse. Dendriform pulmonary ossification is a rare form of diffuse heterotopic bone formation within the lungs.[11] Usually unrecognized radiographically while the patient is alive, it typically is diagnosed at postmortem examination. In chronic pulmonary fibrosis, branching spicules of bone extend through the lung interstitium in a racemose or dendriform manner.[68] Such calcification often is invisible on chest radiographs; HRCT using appropriate window settings shows tiny calcific deposits in the lung periphery.[10–11]

High-attenuation consolidation and lung masses may be the result of a variety of drugs, iatrogenic and idiopathic causes. The tri-iodinated antiarrhythmic drug amiodarone can cause lung toxicity as iodine is deposited in the lungs. The most common CT findings include septal thickening and interstitial fibrosis. Amiodarone pulmonary toxicity can result in high-attenuation focal or multifocal parenchymal opacities due to incorporation of amiodarone into type II pneumocytes.[69]

Iodinated oil pulmonary embolism may follow transcatheter oil chemoembolization or after lymphangiography. It may give an impression of calcification in CXR. However, HRCT findings consist of multifocal patchy areas of ground-glass attenuation and high-attenuation areas of consolidation and collapse.[70]

**Figure 38:**
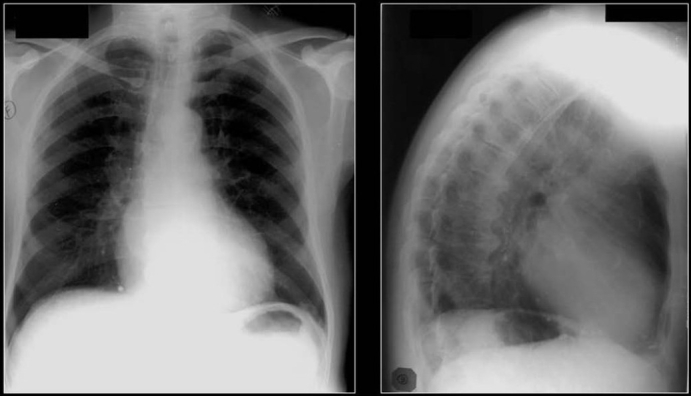
A chest radiograph shows thorotrast deposition in posterior mediastinal lymph nodes seen as tiny metallic nodules. This patient had a carotid angiogram performed in the mid forties for a subarachnoid hemorrhage where thorotrast was used as a contrast agent. The spleen, which is not clearly depicted here also showed lace-like metallic densities due to thorotrast deposition

Thorotrast is a colloidal suspension of thorium dioxide, which was used as an intravascular contrast agent until the 1950s. Thorotrast is retained by the reticuloendothelial system and is found in the liver, spleen, lymph nodes and bone marrow years after it is administered. Thorium is an alpha emitter and has a half-life of 400 years and is carcinogenic. Lymph nodes within the thorax retain the agent and may cause opacities of metallic density within the thorax. The contrast agent is no longer used[71–72] [Figure 38].

## Conclusion

A variety of pathological states can result in calcified and high-attenuation PNs. The imaging evaluation of pulmonary nodules (PN) is based on clinical history, size and appearance of the nodule and feasibility of obtaining a tissue diagnosis. We have discussed the various patterns of calcification in benign and malignant PN and suggest ways to avoid pitfalls when unusual patterns are encountered as calcification in PN as a criterion to determine benign nature is fallacious and can be misleading. The differential considerations of calcified and high-attenuation lung lesions including calcified granuloma, PH, carcinoid and lung metastases or a primary bronchogenic carcinoma have been discussed and presented as a pictorial assay emphasizing the various patterns of calcification in PN to aid diagnosis and to discuss the differential diagnosis and the pathogenesis where it is known.
